# Financial Well-Being Across Age Cohorts and Its Impact on Retirement Security

**DOI:** 10.1093/geroni/igaa057.366

**Published:** 2020-12-16

**Authors:** Stephanie Skees, Stephen Roll

**Affiliations:** 1 Washington University in St. Louis, Saint Louis, Missouri, United States; 2 Washington University in St. Louis, St. Louis, Missouri, United States

## Abstract

The lack of retirement savings in the United States has been well-documented. For example, in 2013 roughly one-third of households near retirement lacked either a defined benefit or defined contribution retirement plan (U.S. Government Accountability Office, 2015), and the median retirement account balance is $0 (Oakley, Brown, & Saad-Lesler, 2018). While this lack of savings is concerning, less clear is the relationship between retirement security and a household’s sense of financial well-being. To that end, this study uses the nationally representative 2017 Survey of Household Economics and Decisionmaking to investigate the relationship between both retirement security indicators and subjective financial well-being—as measured through the Consumer Financial Protection Bureau’s financial well-being scale—across the life course. Specifically, we use multiple regression approaches to compare how financial and knowledge-based indicators of retirement security contribute to the financial well-being of young, mid-career, and pre-retirement cohorts. Preliminary results indicate that feeling “on track” for retirement savings and comfort in making retirement savings decisions were among the strongest contributes to a sense of financial well-being across age cohorts. However, these indicators were particularly important to the financial well-being of older households. Surprisingly, having a 401(k), IRA or pension had no significant impact on savings when controlling for other factors. This study speaks to the importance of both providing effective retirement savings tools, including both educational and financial resources, at multiple points across the life course. It also contributes to a small but growing literature on the intersections between subjective financial well-being and financial decisionmaking.

